# Changes in Onchocerciasis Ov16 IgG4 Rapid Diagnostic Test Results Over One-Month Follow-up: Lessons for Reading Timeframe and Decision-Making

**DOI:** 10.4269/ajtmh.21-1201

**Published:** 2022-08-01

**Authors:** Hugues C. Nana-Djeunga, Capucine M. Sicard, Aude E. Mogoung-Wafo, Cédric B. Chesnais, Hugo Deléglise, Rufine Touka-Nounkeu, André Domche, Allison Golden, Amy D. Klion, Thomas B. Nutman, Michel Boussinesq, Joseph Kamgno, Sébastien D. Pion

**Affiliations:** ^1^ Centre for Research on Filariasis and other Tropical Diseases, Yaoundé, Cameroon;; ^2^ Institut de Recherche pour le Développement, UMI 233-Inserm U1175-Montpellier University, Montpellier, France;; ^3^ Parasitology and Ecology Laboratory, Department of Animal Biology and Physiology, Faculty of Science, University of Yaoundé 1, Yaoundé, Cameroon;; ^4^ Diagnostics Global Program, PATH, Seattle, Washington;; ^5^ Laboratory of Parasitic Diseases, National Institute of Allergy and Infectious Diseases, Bethesda, Maryland;; ^6^ Faculty of Medicine and Biomedical Sciences, University of Yaoundé 1, Yaoundé, Cameroon

## Abstract

The SD Bioline^®^ IgG4 rapid diagnostic test (RDT) detects IgG4 antibodies induced by the *Onchocerca volvulus-*specific antigen Ov16. We evaluated the stability of the RDT results over 1 month, at different time points after completion of each assay, using eluted dried blood spots collected in central Cameroon. Agreement coefficients regarding positivity between 30 minutes and 24 hours, 1, 2, 3, and 4 weeks were, 96.4%, 93.4%, 93.3%, 93.2%, and 93.2%, respectively. Between 30 minutes and 24 hours, 3.6% of the 15,444 tests showed inconsistent results with 81.2% of these tests changing from negative to positive, increasing *O. volvulus* antibody prevalence from 23.9% to 26.2% (*P* < 0.0001). This change from negative to positive outcome was confirmed at the subsequent timepoints. Depending on the desired accuracy of prevalence estimates, reading time may have to be redefined more strictly.

In 2021, the World Health Organization (WHO) officially adopted the 2021–2030 roadmap for neglected tropical diseases (NTDs), designed to end the neglect and attain the sustainable development goals.^1^ The availability of point-of-care diagnostics usable at the community level and in low-resource settings was identified as a core programmatic and cross-cutting technical dimension for the assessment of disease-specific actions to meet the 2030 targets. Ov16 serology by ELISA among children <10 years of age is currently used in decision-making for stopping mass drug administration (MDA) and verifying elimination of human onchocerciasis.^2^ The availability of a point-of-care rapid diagnostic test (RDT) and the development of reagents to facilitate in-country training and support quality assurance greatly enhanced the operational utility of Ov16 serology in mapping, monitoring progression toward elimination, and post-MDA surveillance.^3^ The test procedure is simple and based on the qualitative detection of IgG4 antibodies against the Ov16 antigen. Briefly, 10 µL of whole blood, serum, or plasma is added to the round well of a cassette, and four drops of a diluent buffer is added into the square assay well to initiate lateral flow. The results are available in a rectangular window as test and control lines and are considered valid from 20 minutes to 24 hours after addition of diluent (30 minutes–24 hours if eluted dry blood spots are used). Reading outside of this timeframe may provide unreliable results. To further refine the experimental protocol for a new use of the RDT with dried blood spots (DBS) and compare the results to those following the manufacturer’s recommendations, we evaluated the stability of the results given by the Ov16 immunochromatographic RDT over the course of 1 month using eluted DBS.

Samples used in this study were collected in the Okola health district (Center Region, Cameroon) as part of a project to evaluate the efficacy of a Test-and-Not-Treat (TaNT) strategy to combat onchocerciasis in areas where the disease is hypoendemic and where loiasis is coendemic. The TaNT strategy consists of 1) the identification, using a rapid test, of those few subjects who harbor high *Loa loa* microfilaremia associated with a risk of postivermectin serious adverse events (> 20,000 mf/mL), 2) the exclusion of the latter from treatment, and 3) the safe treatment of the rest of the population with ivermectin.^4^ During the implementation of the TaNT strategy in the Okola health district in 2015, DBS were prepared on filter paper (TropBio Pty Ltd, Queensland, Australia) for 15,444 participants and stored at −80°C. To assess for the presence of Ov16 IgG4 antibodies indicating exposure to onchocerciasis, the SD BIOLINE Onchocerciasis IgG4 rapid diagnostic (Abbott Standard Diagnostics, Inc., Yongin, Republic of Korea) was used. Briefly, one earbud (“petal”) of the DBS sample (∼10 µL of blood) was eluted overnight at ∼4°C in 60 µL of the Ov16 RDT buffer provided by the manufacturer. After adding 5 µL Ov16 RDT buffer to the round sample well of a barcode-labeled cassette, 10 µL of DBS eluate was pipetted into the same sample well, followed by addition of 75 µL of assay diluent into the square well. The test result was read at 30 minutes and then again at 24 hours. A subset of 1,597 cassettes (10.3%) were randomly selected and stored on shelves at room temperature, in a dried environment without humidity, for weekly follow-up of the stability of the results for 4 weeks. The results were reported using a semiquantitative score defined by the relative intensity of test and control lines (Figure 1). Each semiquantitative result was recorded in a database, and a snapshot of each cassette was taken as visual backup of the reading. Prior to testing, each Ov16 cassette lot underwent quality assurance testing as previously described.^5^

**Figure 1.**
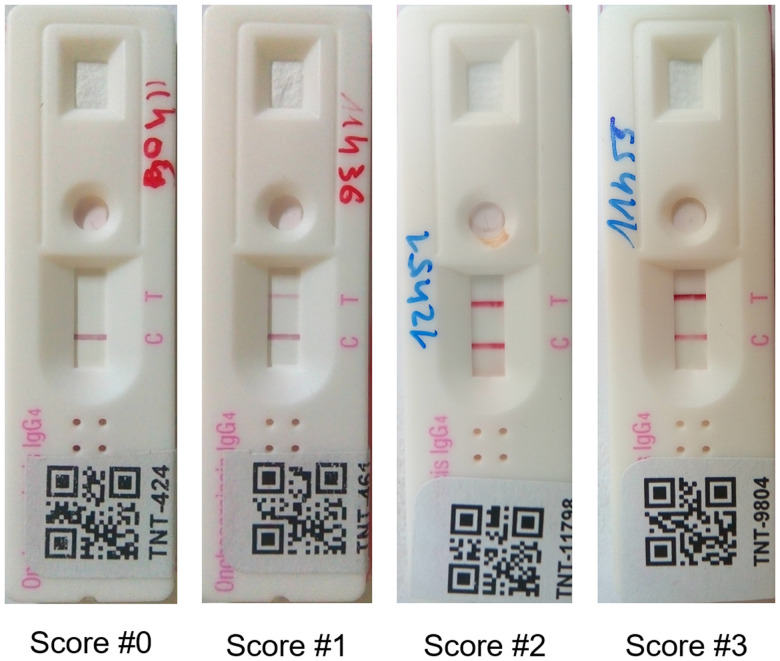
Photographs of Ov16 rapid diagnostic test with the four possible scores. Score #0 for negative tests with no visible test (T) line, Score #1 for a positive test with a T line weaker than the control (C) line, Score #2 for a positive test with a T line approximately as dark as the C line, and Score #3 for a positive test with a T line darker than the C line. This figure appears in color at www.ajtmh.org.

Overall, Ov16 RDT results were consistent in time, with agreement coefficients between 1) 30 minutes and 2) 24 hours, 1, 2, 3, and 4 weeks of 96.4%, 93.4%, 93.3%, 93.2%, and 93.2%, respectively. However, between 30 minutes and 24 hours, 3.6% of the 15,444 tests showed inconsistent results with 81.2% of these tests changing from negative to positive, increasing the observed Ov16-specific antibody prevalence from 23.9% to 26.3% (*P* < 0.0001). Regarding the subsequent timepoints using the subsample of 1,597 cassettes, the proportion of positive tests increased significantly (according to McNemar’s test for matched pairs of observations) from 27.9% at 30 minutes to 30.4% at 24 hours, 31.5% at 1 week (*P* < 0.0001), 32.2% at 2 weeks (*P* < 0.0001), 32.2% at 3 weeks (*P* < 0.0001), and 32.5% at 4 weeks (*P* < 0.0001) (Figure 2). Cuzik’s test confirmed the significance of the trend toward a higher positivity rate with time (*P* = 0.003). This increase reflects a higher proportion of changes from negative to positive than from positive to negative, but changes occurred in both directions. These changes, either from negatives to positives or from positives to negatives, only occurred in tests with a score of 0 or 1 at 30 minutes.

**Figure 2.**
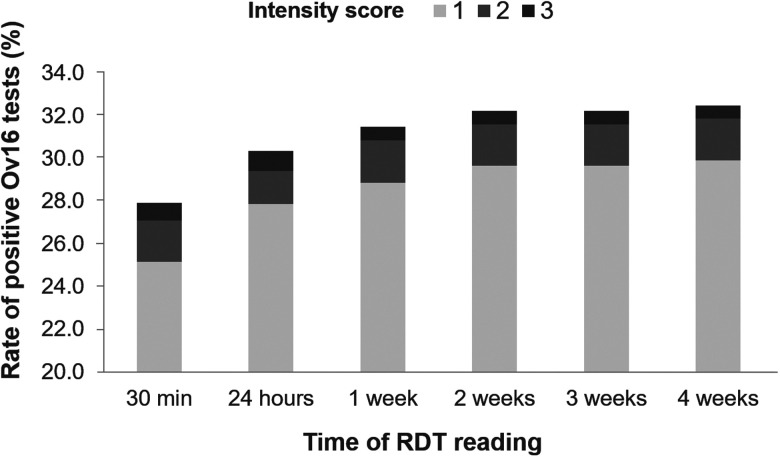
Evolution of Ov16 rapid diagnostic test results over a 4-week follow-up.

We analyzed whether the change in result between 30 minutes and 24 hours was associated with the following individual parameters: age, sex, presence of *L. loa* microfilaremia and presence of *Mansonella perstans* microfilaremia (during the blood collection survey, a 50 µL calibrated blood smear was performed for each participant to quantify *L. loa* and *M. perstans* microfilarial levels).^4^ A logistic regression indicated that the probability of changing from negative to positive between 30 minutes and 24 hours increased with the age of the person tested (reference category: 5–10 years old) and was also higher in individuals with *L. loa* microfilaremia (Figure 3A). A second logistic regression indicated that the probability of reversion of the test from positive to negative tended to be lower in adults than in individuals < 20 years of age but higher for individuals with *L. loa* microfilaremia (Figure 3B).

**Figure 3.**
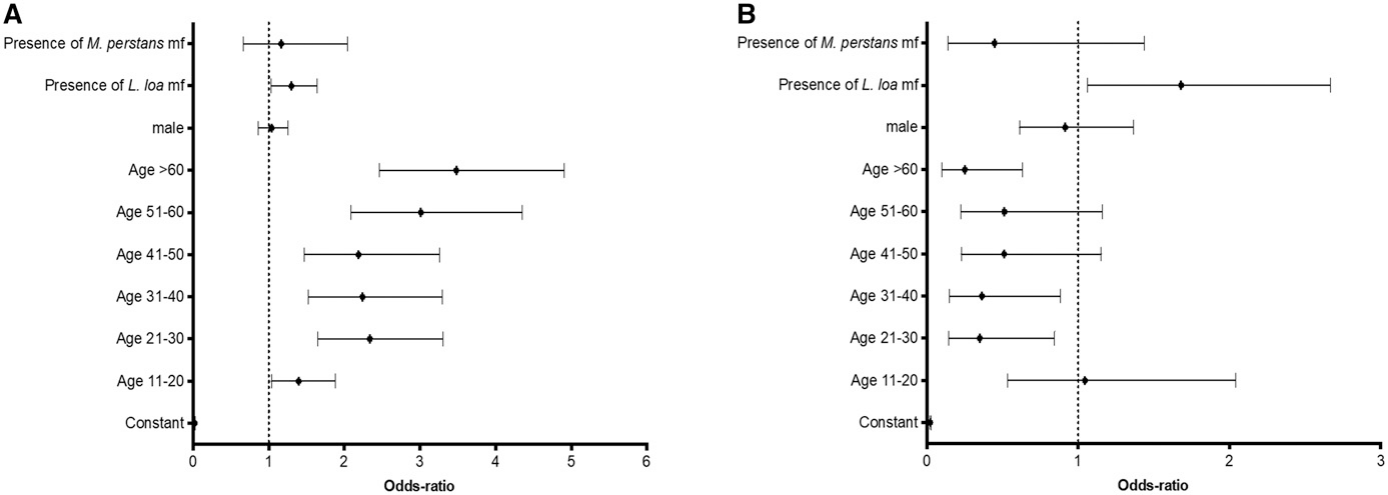
Odds-ratios for changes in Ov16 RDT result between 30 minutes and 24 hours. (**A**) change from negative to positive on the subsample of tests that were negative at 30 minutes; (**B**) change from positive to negative on the subsample of tests that were positive at 30 minutes.

This study was designed to test, on a large scale, the stability of the results provided by the Ov16 RDT over 1 month. The results of this study indicate a small, though statistically significant, difference in Ov16 RDT results between 30 minutes and 24 hours, the boundaries of the recommended window for reading, with a relative increase in positivity of about 10%. The positivity rate subsequently increased linearly for up to 4 weeks, reaching a relative increase of 35% compared with initial values. However, the fact that most (88.7%) tests positive at 24 hours were already positive at 30 minutes indicates that 30 minutes is sufficient for the assay to react.

Important variations were observed in the outcome of the test among 30 minutes, 24 hours, and 1 week. The manufacturer’s instructions are based on stability of result using whole blood and plasma or sera samples. Previous studies conducted using an experimental prototype, on 100 blinded spiked blood specimens, demonstrated stable, and long-lasting test results.^6^ The changes (or instability) observed in the present study only occurred when the test result at 30 minutes was borderline (intensity of test line was weak compared with the control line or score #1), and could be due to various factors, in particular physicochemical factors such as heat, humidity, or pH that could impact the quality of the test or trigger a decline in the affinity between antibodies and antigens over time. Also, the use of DBS in the current study instead of fresh blood or plasma/serum to perform the test could have affected the results, because red blood cells or their lysed fragments may have altered the chemical or visual separation.^6^^,^^7^ Results using this experimental protocol for running DBS on the Ov16 rapid tests suggest that the earliest read should be later than in the current study (1 hour rather than 30 minutes) for the sample types listed in the manufacturer’s instructions for use.^8^

We observed that the probability of a negative test to change to positive was higher in older individuals. The effect of age on the probability of switching from negative to positive may be explained by the presence of higher concentrations or a greater diversity of antibodies after a lifetime of exposure to different pathogens. Also, the effect of *L. loa* microfilaremia on the probability of a changing result between 30 minutes and 24 hours, was significant but inconclusive because it affected the change in both directions. *L. loa* has previously been incriminated in the cross-reaction of RDTs for lymphatic filariasis, with a clear dose-effect relationship between the density of *L. loa* microfilaremia and the probability of the antigen-detecting RDT to react.^9^^–^^11^ However, the Ov16 RDT was not designed to detect antigens but specific antibodies and has not been found to be cross-reactive with *L. loa* when used in an IgG4-based format.

Ov16 RDT has been proposed as a surveillance tool for postcontrol periods to support onchocerciasis control programs. This test is particularly informative when it is negative, to prove that a population has not been exposed to *Onchocerca volvulus*. This study did reveal notable changes in the outcome of Ov16 RDT, when used with eluted DBS, between 30 minutes and 24 hours, thus confirming the significant reduction of the limit of detection of the targeted recombinant Ov16 IgG4 between 30 minutes (25 ng/mL) and 24 hours (15 ng/mL).^8^ Depending on the desired accuracy of the prevalence estimate (e.g., whether for onchocerciasis elimination mapping or decisions to stop MDA),^3^^,^^12^ the time of reading may have to be redefined more strictly and with evaluation of sensitivity/specificity using well-characterized clinical samples over time. Even though the objective of our study was not to assess the sensitivity/specificity of the Ov16 RDT, and as such we did not perform comparative diagnostic testing on the samples to serve as a reference, it was demonstrated that the sensitivity and specificity of the Ov16 monoplex RDT approached that reported for the gold standard Ov16 ELISA.^13^ The Ov16 ELISA is currently recommended by WHO guidelines for stopping MDA and verifying elimination of human onchocerciasis. However, Ov16 RDT used with eluted DBS presents considerable advantages over the latter: lower limit of detection and higher dilution factor conferring a higher sensitivity, as well as easier use in terms of logistics and number of samples processed per operator and per day.^14^ Therefore, Ov16 RDT using eluted RDT appears to be a viable alternative to ELISA Ov16. Evaluation of the performance of these RDTs prior to their use in stop-MDA assessments has been recommended in the newly released WHO guidelines.^8^ Such a comparative phase to understand the sensitivity and specificity should be included in future research.
